# Targeting gut health: Probiotics as promising therapeutics in alcohol-related liver disease management

**DOI:** 10.3934/microbiol.2025019

**Published:** 2025-06-11

**Authors:** María José Lorenzo Pisarello, Antonela Marquez, Adriana Perez Chaia, Jaime Daniel Babot

**Affiliations:** 1 Centro de Referencia para Lactobacilos (CERELA)-CCT NOA Sur-CONICET, 4000, San Miguel de Tucumán, Tucumán, Argentina; 2 Universidad Nacional de Tucumán, 4000, San Miguel de Tucumán, Tucumán, Argentina; 3 Mayo Clinic, 55901, Rochester, MN, USA; 4 Universidad del Norte Santo Tomás de Aquino, 4107, Yerba Buena, Tucumán, Argentina

**Keywords:** beneficial bacteria, prebiotics, postbiotics, synbiotics, alcohol consumption, dysbiosis, intestinal barrier function, liver inflammation

## Abstract

Alcohol consumption represents a major global health issue, accounting for approximately 4.7% of annual deaths and 5.1% of the disease burden worldwide. The liver is particularly vulnerable to alcohol-related damage, with chronic alcohol use leading to a spectrum of alcohol-associated liver diseases, including fatty liver, alcohol-associated hepatitis, cirrhosis, and hepatocellular carcinoma. Despite public awareness of the risks associated with excessive alcohol intake, a substantial proportion of the global population continues to consume alcohol, contributing to the increased incidence of liver-related conditions. Dysbiosis of the gut microbiota has emerged as a critical factor in the pathogenesis of alcohol-associated liver diseases, as alcohol consumption alters microbial composition and increases intestinal permeability, which contributes to systemic inflammation and liver injury through the translocation of endotoxins. Recent research into the therapeutic potential of probiotics, prebiotics, and synbiotics highlights their ability to restore microbial balance and enhance intestinal barrier function. Studies demonstrate that these interventions can significantly improve liver enzymes and reduce inflammation, suggesting their complementary role in the management of alcohol-associated liver diseases. However, further research is necessary to elucidate optimal dosing strategies and long-term efficacy. This review underscores the importance of a multifaceted approach toward understanding alcohol-associated liver diseases and the therapeutic potential of modulating the gut-liver axis through microbiota-targeted strategies.

## 1. Impact of alcohol consumption on global health

Alcohol consumption is a significant contributor to mortality and health issues worldwide. Epidemiological data indicate that approximately 4.7% of global deaths annually are linked to alcohol use, accounting for around 5.1% of the global disease burden [1]. The liver, in particular, is highly susceptible to the detrimental effects of alcohol, with nearly 50% of deaths due to chronic liver disease being related to alcohol consumption [2]. Research indicates that 31–60 g of daily alcohol consumption increases the risk of developing liver diseases by 10.9% [3]. Despite heightened public awareness of the risks associated with excessive alcohol intake, approximately 2.5 billion individuals globally, aged 15 and older, continue to consume alcohol. The World Health Organization (WHO) further reports that roughly 35% of women and 51% of men around the world consumed alcohol within the past year. On average, individuals aged 15 and older consume approximately 12 g of pure ethanol per day, amounting to around 5.5 L annually [1]. Nonetheless, alcohol consumption habits and the types of beverages consumed vary significantly across different regions. In the Americas and Europe, ethanol intake ranges from 7.5 to 9.2 L per year. Conversely, many North African and Eastern countries report substantially lower alcohol consumption, with some areas having negligible alcohol intake. Preferences for specific alcoholic beverages differ globally; hard spirits constitute the most widely consumed category of alcohol, comprising 45.4%, followed by beer at 35.3% and wine at 12.4% [1]. This consumption pattern shifts considerably by region. In Southeast Asia, hard spirits make up 89.2% of total alcohol consumption, while beer and wine account for only 10.4% and 0.4%, respectively. In contrast, in the Americas, hard spirits contribute to 31.5% of alcohol consumption, with beer and wine representing 53.8% and 13.8%, respectively [1]. Additionally, drinking patterns vary according to income. For example, approximately 70% of alcoholic beverages consumed in countries classified by the World Bank as lower-middle income, like Bolivia and Venezuela, are spirits. In contrast, high-income countries such as Chile, Uruguay, and Spain predominantly favor beer. Argentina, however, shows a preference for beer (43.9%) and wine (37.4%) [1].

The COVID-19 pandemic has further exacerbated the public health situation, significantly influencing individuals' physical and mental well-being. Throughout the pandemic, a notable increase in alcohol consumption occurred, with online alcohol sales in the United States rising by 477% and monthly alcohol sales in Canada experiencing a 38% relative increase [4]. In Argentina, studies indicate a 20% increment in alcohol consumption among young adults aged 18–24, contrasted with a 10% increase among individuals aged 55 and older [5]–[7]. As a result, the rise in alcohol intake is likely to lead to an increase in health-related issues. Therefore, it is critical to explore new treatments aimed at mitigating the negative health effects associated with alcohol consumption.

## 2. Alcohol-related liver diseases: Pathogenesis and clinical implications

Alcohol use disorder (AUD) is responsible for over 200 conditions, including neuropsychiatric disorders, chronic diseases, cancers, accidents leading to permanent disability, and liver damage. According to the WHO, alcohol was responsible for approximately 2.6 million deaths—equivalent to 4.7% of total deaths worldwide—in 2019. Additionally, alcohol contributes to 4.6% of disability-adjusted life years (DALYs), which represent the burden of disease attributable to alcohol consumption under the assumption that these DALYs would not have occurred in the absence of alcohol use [1]. Notably, chronic alcohol consumption leads to around 2 million liver disease-related deaths each year, with liver cancer accounting for 38.3% and cirrhosis for 61.7%. These diseases disproportionately affect men, who are 2–3 times more likely to be affected than women [8],[9]. A recent comprehensive study revealed that the prevalence of alcohol-related liver disease (ALD) in a random sample of the general population is approximately 3.5% (95% CI, 2.0%–6.0%). In contrast, cohorts diagnosed with AUD exhibit a prevalence of ALD at 55.1% (95% CI, 17.8%–87.4%) and alcohol-related cirrhosis at 12.9% (95% CI, 4.3%–33.2%) [10].

**Figure 1.**
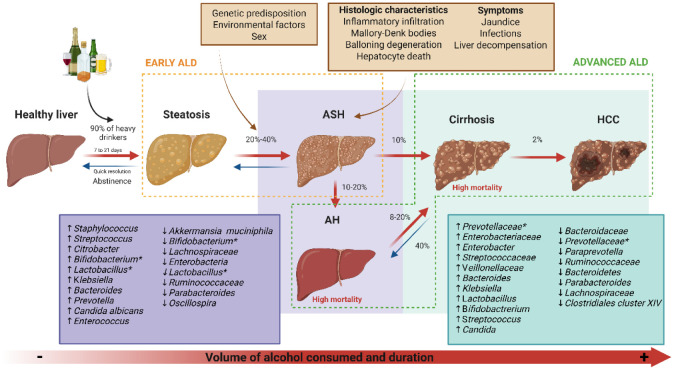
ALD progression and gut microbiota changes. ALD progresses through several stages: from steatosis to fibrosis, cirrhosis, and potentially HCC. Alcohol exposure alters the intestinal microbiota both in quantity and diversity, leading to dysbiosis that persists throughout the disease's progression, regardless of the presence of inflammation. The red arrows illustrate disease progression, while the blue arrows indicate reversibility. Genera marked with an asterisk (*) lack consensus regarding their counts' alteration in the literature. The violet box indicates stages with hepatitis, while the green box indicates those with fibrosis.

ALD encompasses a spectrum of liver disorders resulting from chronic alcohol consumption, including alcohol-associated hepatitis (AH), steatosis (fatty liver), alcohol-associated steatohepatitis (ASH), alcohol-associated fibrosis, cirrhosis, and hepatocellular carcinoma (HCC) in some cases [11]. It should be noted that not all individuals who consume excessive alcohol will develop clinically significant ALD; evidence shows that approximately 10%–20% of chronic heavy drinkers will progress to severe liver conditions such as AH or cirrhosis [12]. The likelihood of developing severe liver disease is influenced by several factors, including genetic predisposition, sex, and environmental factors [5]. Approximately 90% of individuals who engage in heavy alcohol use develop fatty liver within 2–3 weeks (Figure 1), a condition that typically resolves quickly with abstinence [13]. However, prolonged alcohol consumption may lead to hepatic inflammation (ASH) in nearly one-third of patients with steatosis, marked by inflammatory cell infiltration (including neutrophils), Mallory–Denk bodies, ballooning degeneration, and hepatocyte death, and characterized by symptoms such as jaundice, infections, and liver decompensation. Additionally, the progression from AH to cirrhosis occurs in only 8%–20% of cases annually, and both conditions are associated with significantly elevated mortality rates. The incidence of HCC among cirrhotic patients is estimated to be around 2% [14]. The risk of advancing through the ALD spectrum increases substantially with both the volume and duration of alcohol consumption, emphasizing the critical role of abstinence in improving liver health. ALD can be categorized into two primary stages: early or asymptomatic ALD (including fatty liver and ASH) and advanced ALD (characterized by AH and cirrhosis, and complications such as ascites, portal hypertension-related bleeding, hepatic encephalopathy, sepsis, multiorgan dysfunction, and HCC) [15],[16].

Despite considerable progress in the understanding of ALD, its pathogenesis remains only partially elucidated. Several hypotheses attempt to explain the mechanisms behind ALD development. Ethanol metabolism occurs primarily in the liver, involving gastric mucosal cells and intestinal microbiota. The main pathways for converting ethanol to acetaldehyde include [17]:

Alcohol dehydrogenase (ADH): This cytosolic enzyme catalyzes the conversion of ethanol to acetaldehyde, simultaneously converting reduced nicotinamide adenine dinucleotide (NADH) to its oxidized form (NAD+).
Catalase: Present in peroxisomes, this enzyme reduces hydrogen peroxide (H_2_O_2_) to water and is also involved in ethanol metabolism.
Cytochrome P450 2E1: This microsomal enzyme, which is upregulated with excessive alcohol intake, produces reactive oxygen species (ROS) such as superoxide anion and hydrogen peroxide.

With chronic alcohol consumption, elevated acetaldehyde levels disrupt the NAD+/NADH ratio and damage mitochondria due to ROS, inhibiting the β-oxidation of fatty acids and resulting in steatosis. Fatty liver, along with inflammation and fibrosis, is recognized as the histological hallmark of ALD, arising from a cascade of events triggered by continued alcohol exposure [18]. Steatosis may also be exacerbated by increased lipogenesis and decreased fatty acid oxidation [19],[20]. Moreover, lipopolysaccharides (LPS) derived from the gut are critical in the development of liver steatosis, inflammation, and fibrosis [21]. The liver is continuously exposed to bacterial components and gut metabolites through the portal vein. This exposure activates several hepatic cells, including Kupffer cells, neutrophils, hepatocytes, sinusoidal endothelial cells, and stellate cells, leading to the secretion of inflammatory mediators such as tumor necrosis factor-alpha (TNF-α) and interleukin-6 (IL-6), which contribute to liver injury and disease progression [22]. Dysregulation of the gut barrier, reflected in increased intestinal permeability, is a vital factor in ALD pathogenesis, exacerbated by dysbiosis [23]. Furthermore, alterations in the gut microbiome may facilitate the translocation of LPS from gram-negative bacteria into the portal circulation, worsening liver inflammation and damage [23]–[26].

In conclusion, the complex pathogenesis of ALD requires a multifaceted approach to understand its development, progression, and potential therapeutic strategies.

## 3. Conventional treatments for alcohol-related diseases

Traditional treatments for ALD typically focus on managing symptoms, halting progression, and preventing complications. They can be broadly categorized into pharmacological treatments, lifestyle interventions, and sometimes liver transplantation. Among the pharmacological treatments, corticosteroids are used to reduce inflammation in severe AH, although their efficacy is limited, especially in cases with poor liver function. Pentoxifylline is sometimes used to treat AH as it helps reduce liver inflammation and fibrosis. N-Acetylcysteine (NAC) is an antioxidant used in some cases to reduce liver damage [27]. Liver transplantation may be considered in severe cases of ALD, particularly if the liver is irreversibly damaged. Regarding lifestyle modifications, alcohol cessation is the most important aspect of the treatment, since continuing alcohol use exacerbates liver damage. Dietary changes are critical, especially in cases of alcohol-induced malnutrition, as well as weight management, addressing comorbid conditions like obesity or fatty liver disease [28]. However, these treatments have some limitations: The absence of universally effective pharmacotherapy for ALD, the side effects of corticosteroids (e.g., increased risk of infections, gastrointestinal bleeding) that can limit their use, and the challenge of maintaining alcohol cessation in patients with alcohol dependence [29].

While conventional ALD treatments focus primarily on symptom management and limiting liver damage, microbiota-targeted therapies offer a novel avenue that could address underlying mechanisms contributing to ALD progression. These strategies have the potential to complement or even replace some traditional treatments, offering a more personalized, microbiome-focused approach to treating ALD [30],[31]. However, more research is needed to fully establish their clinical effectiveness and long-term benefits [32]. Some differences between conventional ALD treatments and microbiota-based therapies are listed in Table 1.

**Table 1.** Differences between conventional ALD treatments and microbiota-targeted therapies.

	Conventional ALD treatments	Microbiota-targeted therapies
Mechanism of action	Primarily aims at reducing inflammation and liver damage	Targets gut-liver axis, restores microbiota balance
Types of treatment	- Abstinence from alcohol - Nutritional support - Corticosteroids - Pentoxifylline - Liver transplantation	- Probiotics - Prebiotics - Fecal microbiota transplantation (FMT) - Selective antibiotics - Dietary interventions
Efficacy	Effective in early-stage ALD (e.g., corticosteroids for AH) but limited in severe cases (advanced liver cirrhosis)	Promising in early-to-moderate ALD stages, but more research is needed for widespread use
Side effects	Corticosteroids: Increased infection risk, weight gain, immunosuppression Pentoxifylline: Gastrointestinal issues, headaches	Probiotics and prebiotics are generally well-tolerated, but safety data for long-term use is sparse
Safety	Side effects associated with long-term use of corticosteroids and other drugs	Probiotics and prebiotics are considered safe; FMT is less standardized and has potential safety concerns
Treatment cost	Often expensive, especially in the case of liver transplantation	FMT is costly and not widely available, probiotics and prebiotics are relatively affordable
Evidence base	Well-established but with limited effectiveness, especially in advanced disease	Emerging field, with clinical trials ongoing to better understand their role in ALD management

## 4. Role of gut microbiota in alcohol-related liver diseases

The human gastrointestinal tract harbors a vast community of over 100 trillion microorganisms, including bacteria, fungi, viruses, and archaea, collectively termed the gut microbiota (GM). In a symbiotic relationship, the GM performs essential functions for the host, including facilitating nutrient extraction, regulating hormonal pathways through the production of microbe-derived metabolites, metabolizing drugs and xenobiotics, and modulating the immune response. While the GM is commonly regarded as a homogeneous entity, significant compositional variation exists across different gastrointestinal tract regions—from the oral cavity to the colon—largely influenced by environmental factors such as pH and oxygen availability. Thus, the GM is dynamic, exhibits considerable inter-individual and intra-individual variability over time, and is influenced by dietary habits, physical activity levels, medication use, and circadian rhythms [33].

The enterohepatic circulation communicates the liver with the intestinal microbiota. Most venous blood from the small and large intestines drains into the portal vein, which carries enterally absorbed nutrients—such as carbohydrates, lipids, and amino acids—along with microbial metabolites to the liver. However, the intestinal mucosa barrier, characterized by tight and adherens junctions on endothelial cells, effectively prevents the translocation of viable bacteria from the lamina propria into the portal circulation. Consequently, the liver is the primary organ that encounters these microbial-derived metabolites, which are crucial for its immune and metabolic functions. Furthermore, bidirectional communication exists between the gut and the liver, since the gut receives metabolites, products, and inflammatory mediators derived from the liver through systemic circulation. This intricate interaction between the gut and the liver is foundational to what is referred to as the gut-liver axis, emphasizing the anatomical and functional connectivity between these two organs [34].

The integrity of the intestinal mucosal barrier is compromised in the context of liver damage, allowing intestinal microbiota and their metabolites to enter the liver as antigenic stimuli. This may induce inflammatory responses and modulate immune functions. Additionally, the liver plays a crucial role in influencing both the quantity and diversity of the intestinal microbiota by modulating bile acid secretion and the associated signaling pathways that regulate pro-inflammatory cytokines [35],[36].

Recent evidence highlights the role of the gut microbiome in the progression of ALD. Alcohol consumption has been shown to promote intestinal bacterial overgrowth and induce alterations in microbial composition, a phenomenon referred to as “dysbiosis” (Figure 1). This condition is characterized by an increase in the release of endotoxin produced by gram-negative bacteria [37]–[39]. In turn, endotoxin can bind to cells on the intestinal mucosa, causing local inflammation, and translocate to extra-intestinal sites, causing systemic inflammation [40],[41]. Both preclinical and clinical studies have demonstrated that dysbiosis is typically characterized by decreased bacterial diversity, increased abundance of pathobionts (such as *Enterococcus* spp.), and reduced levels of beneficial bacteria (such as *Bifidobacterium* spp. and *Akkermansia muciniphila*). For example, stool analyses from individuals with ALD revealed decreased fungal diversity alongside an increase in *Candida albicans* [42],[43]. A study examining stool samples from patients with AUD indicated an elevated prevalence of *Enterobacteriaceae* and *Lactococcus* phages in individuals with more progressive liver disease [44]. Conversely, a higher abundance of *Lactococcus* phages was also observed in patients with metabolic-associated steatotic liver disease (MASLD) who consumed minimal amounts of alcohol [45]. Another investigation found that phages from *Escherichia*, *Enterobacteriaceae*, and *Enterococcus* were overrepresented in stools of patients with AH, while *Parabacteroides* phages were significantly underrepresented [46]. As previously noted, fecal levels of *A. muciniphila* were significantly lower in patients diagnosed with ASH in comparison to healthy controls. Notably, treatment of alcohol-fed mice with *A. muciniphila* conferred protection against alcohol-induced gut permeability and enhanced the thickness of the mucus layer along with tight junction protein expression [47]. Likewise, research by Leclercq et al. (2014) demonstrated that alcohol-dependent patients exhibiting high intestinal permeability—with no accompanying fibrosis or cirrhosis—had lower levels of *Ruminococcus*, *Faecalibacterium*, *Subdoligranulum*, *Oscillibacter*, and *Anaerofilum* compared to control subjects. Following a 3-week detoxification program, these authors observed an increase in the relative abundance of *Ruminococcus* and *Subdoligranulum*, alongside elevated levels of *Bifidobacterium* spp. and *Lactobacillus* spp. [48]. In additional studies, bacterial endotoxin and peptidoglycan concentrations, alongside Toll-like receptor (TLR) expression in peripheral blood mononuclear cells, showed a decrease during detoxification among non-cirrhotic, alcohol-dependent participants [49]–[51]. Furthermore, even a brief 5-day treatment involving *B*. *bifidum* and *Lactobacillus plantarum* 8PA3, combined with standard therapy (abstinence and vitamin supplementation), resulted in a notable increase in bifidobacteria and lactobacilli counts and a more rapid reduction in elevated transaminase levels compared to standard therapy alone [52]. The diversity of viruses, particularly mammalian viruses, was also found to increase in the fecal samples of patients with AH (for a detailed overview, see also [53]).

Similar to MASLD, dysbiosis of the GM in ALD is considered a potential risk factor for the progression of liver disease. The depletion of beneficial short-chain fatty acid (SCFA)-producing bacteria such as *Lactobacillus*, *Bifidobacterium*, and *Faecalibacterium prausnitzii* leads to a reduction in luminal levels of butyrate, propionate, and acetate [34],[54]. SCFAs, particularly butyrate, serve as primary energy substrates for colonocytes and regulate intestinal barrier integrity through enhancement of tight junction protein assembly and inhibition of histone deacetylases (HDACs), thereby modulating gene expression related to epithelial integrity and inflammation [55],[56]. The loss of SCFAs weakens epithelial tight junctions, increases paracellular permeability, and facilitates translocation of pathogen-associated molecular patterns (PAMPs), such as LPS and peptidoglycan, into the portal circulation [48]. These microbial products activate hepatic TLRs, particularly TLR4 on Kupffer cells, initiating MyD88- and TRIF-dependent signaling cascades that culminate in nuclear translocation of NF-κB and the secretion of pro-inflammatory cytokines such as TNF-α, IL-1β, and IL-6 [51],[57],[58]. In parallel, microbial metabolism of dietary choline into trimethylamine (TMA) and its subsequent hepatic conversion to trimethylamine N-oxide (TMAO) via flavin-containing monooxygenase 3 (FMO3) is perturbed in ALD. Ethanol-associated dysbiosis increases TMA production while suppressing FMO3 expression, resulting in elevated circulating TMA levels with potential cardiometabolic and pro-inflammatory effects [59]. Moreover, chronic alcohol consumption alters microbial and host bile acid metabolism. Ethanol disrupts enterohepatic circulation by impairing FXR and TGR5 signaling pathways, both of which are central to maintaining metabolic and immunological homeostasis. FXR activation represses hepatic CYP7A1 via induction of fibroblast growth factor 19 (FGF19), while TGR5 activation on Kupffer cells attenuates LPS-induced inflammatory signaling via cAMP-mediated suppression of NF-κB [60],[61]. Dysbiosis-driven deconjugation and dehydroxylation of primary bile acids increase the proportion of hydrophobic secondary bile acids, which exacerbate hepatocyte apoptosis and cholestatic injury [62]. These alterations contribute to hepatic lipid accumulation and inflammation, further promoting liver injury [63]. Additionally, microbial-derived neurotransmitters—such as serotonin, dopamine, and γ-aminobutyric acid (GABA)—further contribute to gut barrier dysfunction and immune dysregulation in ALD. Elevated intestinal serotonin levels, driven by bacterial metabolism of tryptophan, disrupt epithelial integrity via 5-HT receptor-mediated signaling, enhancing intestinal permeability and promoting systemic inflammation [64]. Dysregulation of gut-derived GABA, produced via bacterial decarboxylation of glutamate, has been associated with altered central nervous system signaling and increased vulnerability to alcohol-seeking behaviors, suggesting a bidirectional gut-brain-liver axis in ALD pathophysiology [65].

In summary, evidence suggests that abstaining from alcohol, the primary therapeutic intervention for ALD, leads to rapid changes in the composition of the intestinal microbiota and improvements in intestinal barrier function. Furthermore, the administration of selected bacteria appears to support the restoration of the protective mucosal barrier and alleviate liver damage induced by endotoxins and may complement conventional therapy. Several ongoing clinical trials are investigating the modulation of the intestinal microbiota in the context of chronic ALD, aiming to enhance recovery in patients with early-stage ALD and stabilize those with cirrhosis. These trial results are expected to yield valuable insights guiding future probiotic interventions for ALD and MASLD.

## 5. Emerging approaches for the treatment of liver diseases

Research into GM and its impact on health and disease has attracted significant attention in recent years. The GM plays an essential role in developing and maintaining a healthy immune system, especially in managing liver diseases, due to its interaction with the liver through the gut-liver axis [66]–[68]. Currently, lifestyle modifications, including sobriety, dietary changes, increased physical activity, and weight loss, are the main strategies for managing liver diseases [67]. Considering the lack of successful pharmacological interventions in ALD and MALSD, the development of effective strategies to prevent and treat them is an area of great interest.

Considering the connection between the microbiome and liver disease, FMT has been investigated as a promising therapeutic method. This approach involves transferring fecal matter from a healthy donor to a recipient's gastrointestinal system, aiming to restore a balanced microbial community in the gut. Even though FMT has mainly been used to treat recurrent *Clostridioides difficile* infections, recent studies have also examined its potential in addressing various other conditions, including ALD [69]–[71]. FMT may alleviate ALD by several mechanisms, namely the restoration of GM diversity (a more balanced GM can help restore normal metabolic processes), decreased intestinal permeability (and limited movement of harmful substances to the liver), reduction of inflammation (FMT may lower the production of pro-inflammatory cytokines and other molecules that contribute to liver damage in ALD), restoration of intestinal barrier function (potentially enhancing the integrity of the intestinal barrier by encouraging the growth of beneficial bacteria that strengthen mucosal defenses), and modulation of metabolic and pathogenic pathways (a healthier GM might also influence the production of metabolites that can positively affect liver metabolism and immune function) [69],[71]. Animal models of ALD have demonstrated that FMT can improve liver histology, reduce liver inflammation, and restore gut microbial diversity. Clinical studies of FMT in ALD are still limited, but some early-stage trials have shown promising results [69],[71].

One of the main challenges in FMT research is the lack of standardization in protocols, such as donor screening, preparation, and delivery methods. This variability can influence the results. Since the GM is highly individual, there is growing interest in identifying patient characteristics that may predict a positive response to FMT [70],[71]. Although FMT may offer short-term benefits, the long-term effects on the GM and overall health are still not fully understood. Additionally, ethical and regulatory challenges exist regarding the use of FMT, including concerns about the safety of unregulated fecal material and the need for thorough clinical trials to confirm its safety and effectiveness [71].

Recent advancements in omics technologies have opened new avenues for improving the prevention and treatment of ALD. Metagenomic analyses enable the identification of microbial communities and their gene content, offering insights into dysbiosis patterns unique to individual patients [72],[73]. Metabolomic profiling captures the biochemical activity of these microbes, including the production of metabolites that may influence liver inflammation and damage [74]. Integrating these omics approaches provides a comprehensive understanding of host–microbiome interactions, laying the foundation for developing personalized microbiome-based therapies tailored to each patient's microbial and metabolic profile [72],[75]. Such precision approaches may enhance treatment outcomes, reduce variability in the responses, and contribute to more effective strategies in managing ALD [73],[74]. Despite their promise, metagenomics and metabolomics approaches face several limitations that may hinder their clinical application in ALD. Metagenomic analyses, particularly 16S rRNA sequencing, often lack the resolution to detect strain-specific microbial variations relevant to disease progression and may be affected by contamination from host DNA and technical biases during sequencing [76]. Whole metagenome shotgun sequencing provides higher resolution but requires significant computational resources and is costly, limiting its routine use [77]. Metabolomic profiling also presents challenges in the identification and quantification of metabolites due to the vast complexity of the human metabolome and incomplete reference databases [73]. Moreover, integrating multi-omics data into a coherent clinical framework requires standardized protocols, advanced bioinformatics pipelines, and robust validation, which are still under development [76]. These challenges can affect reproducibility, delay the translation of findings into therapeutic strategies, and limit the scalability of personalized interventions in ALD management.

Recent research has shown that the modulation of the GM by probiotics, prebiotics, and postbiotics may protect the liver through the gut-liver axis. This approach is being explored as a potential strategy to prevent and improve the adverse effects of major liver conditions such as cirrhosis and alcohol-associated and non-alcoholic steatosis [66]–[78]. Probiotics are defined as “live microorganisms which when administered in adequate amounts confer a health benefit on the host” [79]. They have been recognized as an alternative strategy for managing digestive and immune health and are increasingly recommended as effective therapeutic interventions for non-communicable diseases, including liver diseases. For microorganisms to be classified as probiotics, they must meet several criteria: they need to be sufficiently characterized and identified through scientifically based methods, proven safe for the intended use, supported by at least one positive human clinical trial, and remain viable at an effective dose throughout their shelf life [80],[81]. Most microorganisms used as probiotics, both in research and commercial development, belong to a limited range of genera, which mainly include *Lactobacillus* spp. and *Bifidobacterium* spp., although other bacteria and certain yeasts are also used [81]–[84]. It should be emphasized that probiotic effects are strain-dependent; different strains of the same species may induce varying responses, thus requiring individual study [79]. Extensive scientific evidence supporting the beneficial properties of specific microorganism strains is driving the growing global demand for probiotics.

A prebiotic is “a substrate that is selectively utilized by host microorganisms conferring a health benefit” [85]. In addition to being non-pathogenic and non-toxic, prebiotics must be resistant to gastric acidity, fermented by gastrointestinal microorganisms, and selectively stimulate beneficial GM. The most commonly used prebiotics are dietary fibers, which are composed of carbohydrates, such as fructo-oligosaccharides, galacto-oligosaccharides, lactulose, and inulin [82],[83],[85]. These dietary fibers resist hydrolysis by human digestive enzymes in the small intestine but can be fermented by colonic microbiota bacteria. Lactulose, a synthetic disaccharide, is the primary prebiotic used in the treatment of hepatic encephalopathy, a common complication of decompensated cirrhosis. It is fermented mainly by colonic bacteria into SCFAs, which are among the most extensively studied fermentative products of prebiotics produced by the microbiota [78].

Furthermore, probiotics and prebiotics can be combined into formulations called synbiotics to enhance the beneficial effect of each component [86]. These act in synergy and ensure the growth and viability of the beneficial organisms in the intestinal tract. This approach, particularly the use of pro-anthocyanidins as prebiotics in conjunction with various probiotics, is considered a promising strategy. Studies indicate that pro-anthocyanidins and probiotics in a symbiotic state are more effective in reducing de novo lipogenesis and stimulating beta-oxidation of fatty acids in fatty liver disease [87].

Recent research has increasingly focused on postbiotics, which are “preparations of inanimate microorganisms and/or their components that confer a health benefit on the host” [88]. This term also includes paraprobiotics, defined as “inactivated microbial cells (non-viable) that confer a health benefit to the consumer” [89]. Postbiotics encompass a plethora of elements, such as SCFAs, peptides, enzymes, vitamins, exo- and endo-polysaccharides, cell wall components, bacteriocins, and other bioactive metabolites [83],[84],[88]. SCFAs such as butyrate, acetate, and propionate are among the most well-known postbiotics. Compared to probiotics, postbiotics offer several advantages: They generally exhibit enhanced safety (reduced risk of sepsis and antibiotic resistance) and provide technological and practical benefits (longer shelf life, since the cold chain is not required for viability). Additionally, postbiotics retain their bioactivity when administered alongside antibiotics or antifungal agents. Therefore, non-viable agents present a safer alternative to live microorganisms, particularly for immunocompromised individuals, such as ill patients or the elderly [84],[89],[90].

Table 2 summarizes the main differences between probiotics, prebiotics, synbiotics, and postbiotics.

Over the past few decades, probiotics, prebiotics, and postbiotics have made important advances in several disease treatments, particularly gastrointestinal disorders such as ALD and MALSD, which are leading causes of chronic liver disease worldwide. Probiotics, prebiotics, and postbiotics were recognized as feasible strategies and have become important alternatives and complementary therapies for promoting health and slowing the progression of these conditions [66]–[68],[78],[90]–[92]. Comprehending their effects and mechanisms, as well as advancing in their clinical applications, constitutes a critical focus area in biomedical research.

The potential mechanisms responsible for the beneficial effects of probiotics are described by the International Scientific Association for Probiotics and Prebiotics (ISAPP). These mechanisms range from broadly conserved among probiotics to exceptionally unique. General mechanisms associated with effects observed across all taxonomic groups include the regulation of GM, inhibition of potential pathogens, regulation of gastrointestinal transit, production of SCFAs and vitamins, improvement of intestinal epithelial barrier function, modulation of the immune response, alteration of hepatic lipid metabolism, and regulation of bile salts metabolism. Less common effects include impacts on the brain-gut axis and consequent neurological and endocrine functions, as well as the production of specific bioactive substances [79],[81],[84]. Claims of such benefits can only be substantiated for strains or substances where the underlying mechanisms have been demonstrated.

Prebiotics selectively stimulate the growth and activity of beneficial gut bacteria, prevent pathogen colonization, regulate the immune system, enhance gut barrier function, and can even influence brain function [82],[84],[85]. Postbiotics exert their beneficial effects through several mechanisms similar to those of probiotics and prebiotics; they promote the growth of beneficial microorganisms, inhibit harmful bacteria colonization, increase adhesion to the intestinal mucosa, improve the epithelial barrier, modulate the immune response, and exhibit antioxidant and anti-inflammatory properties. In addition, some postbiotics, like SCFAs, can also act as signaling molecules that influence host metabolism [84],[88].

**Figure 2.**
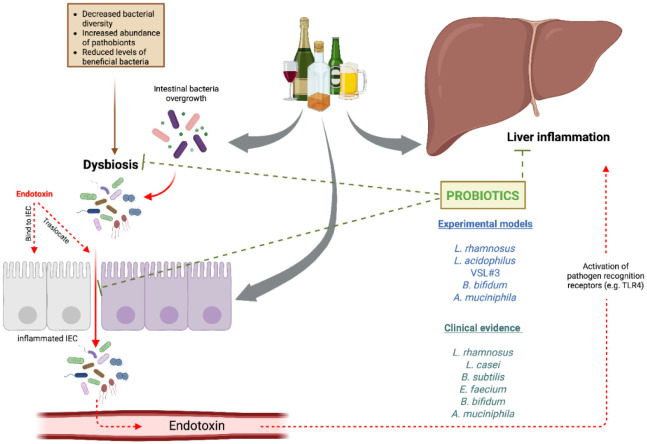
Effects of probiotics in ADL. Ethanol contributes to liver injury through multiple mechanisms, including GM modulation, disruption of the intestinal barrier, and increased levels of pro-inflammatory cytokines. Probiotics have emerged as a therapeutic option to mitigate these detrimental effects, demonstrating efficacy in both clinical studies and experimental models of ALD.

Among the aforementioned mechanisms of action of probiotics, prebiotics, and postbiotics, the main mechanisms highlighted in recent studies on metabolic and hepato-gastrointestinal disorders include modulation of GM composition, enhancement of gut barrier function, and regulation of inflammatory response [67],[83],[91] (Figure 2).

**Table 2.** Differences between probiotics, prebiotics, synbiotics, and postbiotics.

	Probiotics	Prebiotics	Synbiotics	Postbiotics
Definition	Live microorganisms, which, when administered in adequate amounts, confer a health benefit on the host	A substrate that is selectively utilized by host microorganisms, conferring a health benefit	A mixture comprising live microorganisms and substrates selectively utilized by host microorganisms that confers a health benefit on the host	Preparations of inanimate microorganisms and/or their components that confer a health benefit on the host
Examples	Mainly *Lactobacillus (casei*, *fermentum*, *johnsonii*, *plantarum*, and *rhamnosus)* and *Bifidobacterium* (*animalis*, *adolescentis*, *bifidum*, *breve*, *infantis*, and *longum*), although other bacteria and certain yeasts, such as *Saccharomyces boulardii*are also used	Dietary fibers, which are composed of carbohydrates such as fructo-oligosaccharides, galacto-oligosaccharides, lactulose, and inulin	Complementary: Prebiotic + Probiotic Synergistic: Live microorganism + Substrate	SCFAs, peptides, enzymes, vitamins, exo- and endo-polysaccharides, cell wall components, bacteriocins, and other bioactive metabolites
Mechanism of action	Regulation of GM; inhibition of potential pathogens; regulation of gastrointestinal transit; production of SCFAs and vitamins; improvement of intestinal epithelial barrier function; modulation of the immune response; alteration of hepatic lipid metabolism; regulation of bile salts metabolism	Stimulation of the growth and activity of beneficial gut bacteria; modulation of SCFA production; bile salt metabolism; prevention of pathogen colonization; immune system regulation; enhanced gut barrier function	The complementary approach combines prebiotics (targeting beneficial autochthonous microorganisms) and probiotics; meanwhile, the synergistic approach selects substrates that are utilized by the co-administered live microorganisms, enhancing their effectiveness	Beneficial microorganism growth promotion; harmful bacteria colonization inhibition; increases adhesion to the intestinal mucosa; epithelial barrier improvement; modulation of the immune response; antioxidant and anti-inflammatory properties. Some postbiotics, like SCFAs, can also act as signaling molecules that influence host metabolism
Health benefits	Improve digestive health (balance GM, combat Infectious diarrhea, reduce antibiotic-related diarrhea, reduce irritable bowel syndrome symptoms), strengthen the immune system, prevent infections, reduce inflammation, alleviate metabolic disorders	Improved gut health (support for beneficial gut bacteria, enhanced digestion), immune modulation, prevents allergies and inflammation	Support healthy GM, improve immune system, enhance nutrient absorption, reduce the risk of chronic diseases (obesity, metabolic syndrome, NAFLD, and ALD)	Improved gut barrier function, modulation of GM, increased antimicrobial defense, anti-inflammatory effects, effects on the gut-brain axis, support for digestive disorders
Source	Probiotic supplements, fermented dairy products (yogurt, kefir, cheese), fermented vegetables (sauerkraut, kimchi, pickles), fermented beverages (kombucha), etc.	Fruits and vegetables, whole grains, legumes (lentils, soybeans), nuts and seeds (almonds, chia seeds), inulin-rich foods (like Jerusalem artichokes), fiber supplements, etc.	Synbiotic supplements, fermented foods with added fiber (yogurt and kefir with prebiotic ingredients, miso soup with prebiotic vegetables)	Postbiotic-rich foods, fermented foods, high-fiber foods, bacterial lysates, cell wall fragments, functional proteins, etc.

## 6. Probiotics, prebiotics, and synbiotics as a therapeutic approach in alcohol-related liver disease

The primary goal in the management of ALD is to restore normal liver function. Achieving this objective necessitates significant lifestyle changes, predominantly the cessation of alcohol consumption. Additionally, the treatment of ALD must be tailored to the severity and specific complications of the disease, which may include dietary modifications, vitamin supplementation, sodium restrictions, procedural interventions to manage portal hypertension, diuretic therapy, medications to treat hepatic encephalopathy, and anti-inflammatory agents [93],[94].

Notably, recent studies have highlighted the potential benefits of probiotics in the context of ALD. Conversely, the effectiveness of postbiotics in relieving the detrimental effects of alcohol consumption has yet to be studied. Probiotics have been shown to target several pathophysiological mechanisms that contribute to the progression of liver damage. Researchers have concentrated on various strategies to modify intestinal microbiota in ALD. Figure 2 summarizes key investigations into the modulation of the GM's impact on the microbiota-gut-liver axis in this disease. An early study exploring the effects of probiotics on alcohol-induced liver injury was conducted by Nanji et al. (1994) [95]. In this experimental model, rats received alcohol and/or *Lactobacillus* GG, which led to a substantial reduction in serum endotoxin levels and an improvement in fatty liver. Likewise, Marotta et al. (2005) demonstrated that supplementation with *Lactobacillus* sp. prevented endotoxemia and liver damage in a model of acute alcohol exposure combined with pancreatitis [96]. Forsyth et al. (2009) further observed reductions in markers of oxidative stress and inflammation in the liver and intestine, accompanied by preserved gut barrier function due to probiotic treatment [97]. Investigations into the molecular mechanisms underpinning these effects revealed that *L*. *rhamnosus* GG culture supernatant inhibits miR-122a, leading to increased occludin expression and protection against ALD in mice [98]. Previous studies showed that TNF-α mediates occludin expression in the intestine via miR-122a regulation [99], suggesting that these probiotic actions reinforce intestinal barrier integrity and mitigate endotoxemia. Moreover, the upregulation of hypoxia-inducible factor and IL-22 by *Lactobacillus* GG in chronic and binge drinking models has been shown to support the regeneration of intestinal epithelial barrier functions [100],[101]. Several probiotic strategies have been validated for their capacity to improve intestinal barrier function in individuals with AUD, primarily utilizing *Lactobacillus*, *B. bifidum*, or *Akkermansia* species [47],[102]. Notably, the probiotic mixture VSL#3, which includes eight strains predominantly from the *Lactobacillus* and *Bifidobacterium* genera, demonstrated beneficial effects on alleviating liver lesions [103]. The administration of *A. muciniphila* demonstrated a capacity to prevent hepatic injury, steatosis, and neutrophil infiltration in murine models of ALD [47]. Combined treatment with probiotics and glutamine in alcoholic rats markedly reduced elevated serum IL-6 and TNF-α levels, suggesting a role in attenuating hepatic inflammation through the suppression of inflammatory cytokines [104]. Mutlu et al. (2009) also reported alterations in mucosa-associated microbiota in rats subjected to high-alcohol diets, which were ameliorated by either *Lactobacillus* GG or oat supplementation [105]. In a pilot clinical study, Kirpich et al. (2008) observed that supplementation with *B*. *bifidum* and *L*. *plantarum* restored the balance of GM and improved liver enzyme levels in individuals with alcohol dependency [52]. A randomized controlled multicenter trial examined the impact of probiotics on patients diagnosed with AH, revealing that oral treatment with *Bacillus subtilis* and *Enterococcus faecium* modulated the microbiota composition, reducing *Escherichia coli* levels and lowering serum LPS levels in comparison to the placebo group [106]. Additionally, the administration of *L. casei* Shirota to patients with alcohol-associated cirrhosis restored neutrophil phagocytic capacity and normalized TLR4 receptor expression, indicating a reduction in inflammatory signals induced by pathogenic ligands [107]. In a pivotal double-blind, placebo-controlled trial, Koga et al. (2013) investigated the effects of *L. casei* Shirota administered via Yakult 400 (Y400) in hospitalized ALC patients over 4 weeks [108]. They observed a significant increase in serum transthyretin levels by week 3 and a decrease in hypersensitive C-reactive protein (hsCRP) by week 4, indicating improved hepatic protein synthesis and reduced systemic inflammation. Moreover, Y400 corrected the imbalance in gut flora characterized by diminished obligate anaerobes and elevated *Enterobacteriaceae*, suggesting restoration of microbial homeostasis. Han et al. (2015) further demonstrated that a 7-day supplementation with *Bacillus subtilis* and *Enterococcus faecium* in AH patients reduced fecal *E. coli* and systemic levels of TNF-α and LPS [106]. Li et al. (2021) conducted a double-blind randomized trial in patients with alcoholic liver injury and found that 60 days of *L. casei* Shirota supplementation significantly decreased triglycerides and LDL cholesterol, while increasing fecal *Lactobacillus* and *Bifidobacterium* counts, highlighting its lipid-lowering and microbiota-modulating effects [109]. Gupta et al. (2022) expanded on these findings in a multicenter, double-blind randomized trial, showing that *L. rhamnosus* R0011 and *L. helveticus* R0052 administered over 7 days to hospitalized AH patients led to reductions in gram-negative bacteria and serum LPS, improved Child–Pugh scores, and decreased ALT and gamma-glutamyl transpeptidase (GGT) levels, alongside favorable shifts in microbial composition, including increased *Bacteroidetes* and decreased *Proteobacteria* and *Fusobacteria* [110]. Most recently, Vatsalya et al. (2023) reported that daily oral administration of *L. rhamnosus* GG in patients with AUD and moderately severe AH reduced liver injury within 1 month and was associated with decreased heavy drinking after six months, suggesting that probiotics may also influence behavioral outcomes [111]. Collectively, these findings support the growing rationale for probiotic therapy as a multifaceted intervention that not only improves gut microbial composition and mitigates hepatic injury but may also modulate alcohol consumption behavior. Ongoing randomized trials (NCT05178069, NCT06607562, NCT03863730, and NCT05007470) continue to investigate the efficacy of various probiotic strains in this clinical context, aiming to establish their therapeutic role in the management of ALD. Recent active or concluded clinical trials are listed in Table 3.

**Table 3.** Concluded and ongoing clinical trials on probiotics and ALD.

Trial number	Location	Title	Treatment	Type	Phase	Status
NCT05178069	USA	*Lactobacillus rhamnosus* GG: A novel probiotic therapy for treating alcohol use disorder	*L. rhamnosus* GG	Interventional	2	Recruiting
NCT06607562	China	Protective effect of probiotics BC99 on liver function in long-term alcohol consumers: A randomized, double-blind, placebo-controlled trial	Probiotics BC99: *Weizmannia coagulans* BC99	Interventional	NA	Recruiting
NCT03863730	Denmark	Profermin®: Prevention of progression in alcoholic liver disease by modulating dysbiotic microbiota: a randomized controlled clinical trial	ReFerm®: Oat gruel fermented with *L. plantarum* 299v	Interventional	NA	Active, not recruiting
NCT05007470	USA	Alterations of the brain-gut-microbiome axis modulates sex differences in outcomes of alcohol-related liver disease	VSL #3	Interventional	NA	Active, not recruiting
NCT01501162	South Korea	Effect of probiotics on gut-liver axis of alcoholic liver disease	*Bacillus subtilis*/*Enterococcus faecium*	Interventional	4	Completed (2012)
NCT02335632	South Korea	Effect of probiotics on gut-liver axis of alcoholic hepatitis	Lacidofil®: *L. rhamnosus* Rosell-11, *L. helveticus* Rosell-52	Interventional	4	Unknown
NCT01922895	USA	Novel therapies in moderately severe acute alcoholic hepatitis	*L. rhamnosus* GG	Interventional	NA	Terminated (2021)

NA: Not applicable.

Prebiotics have been shown to enhance the growth and activity of specific or less prevalent microbial populations within the host's gut. Consequently, they have been investigated for their potential to modify dysbiosis associated with ALD. A recent study demonstrated that dietary fiber intake increased the abundance of *Bacteroides acidifaciens* in a mouse model of ALD, relieving the liver injury [112]. Likewise, research by Ferrere et al. (2017) revealed that treatment with pectin in alcohol-fed mice not only restored levels of beneficial *Bacteroides* but also completely prevented alcohol-induced steatosis and liver inflammation [113]. Moreover, fruit extracts rich in flavonoids exhibited promising effects by modulating the liver-gut axis. In a mouse model subjected to acute ethanol-induced liver injury, these extracts enhanced the intestinal microbiota, elevating populations of *Dubosiella*, *Lactobacillus*, and *Bifidobacterium*, which are implicated in lipid metabolism, oxidative stress management, and iron homeostasis [45]. The interplay between alcohol consumption and enteric dysbiosis also involves the dysregulation of the mucosal innate immune response. For example, the administration of ethanol alongside fructo-oligosaccharides via an intragastric feeding tube restored levels of the antimicrobial peptide Reg3g, leading to a reduction in intestinal bacterial overgrowth. This restoration was associated with improved hepatic biomarkers, including decreased serum levels of aspartate aminotransferase and lowered hepatic triglyceride concentrations [114].

Regarding the exploration of the use of synbiotics for alleviating ALD, mice with alcohol-associated liver disease were administered a synbiotic formulation containing inosine and *A. muciniphila* in a recent study. This treatment led to significant improvements in various health markers, including reductions in alanine aminotransferase and aspartate aminotransferase levels, as well as decreased LPS concentrations. Moreover, the synbiotic intervention significantly diminished the infiltration of inflammatory cells and lipid accumulation in the liver, while also restoring intestinal barrier function [115]. Similar findings were reported by the same research group, which investigated the effects of combining inosine with *L. rhamnosus* GG, further corroborating these beneficial outcomes [116],[117].

Despite growing evidence supporting the therapeutic potential of probiotics in ALD, several important limitations must be acknowledged. A major challenge lies in the variability of individual gut microbiomes, which are shaped by a complex interplay of dietary habits, genetics, lifestyle, and environmental exposures. This intrinsic heterogeneity means that the response to probiotic supplementation can vary widely among individuals, complicating the prediction of clinical outcomes [118],[119]. Preclinical studies often rely on standardized animal models that do not capture this diversity, limiting the translatability of findings to heterogeneous human populations [118]. Furthermore, probiotic efficacy is highly strain-specific; different strains may exert distinct or even opposing effects, and the mechanisms underlying these actions remain incompletely understood [119]. As such, selecting appropriate strains for targeted interventions in ALD is not straightforward. Clinical research to date has been constrained by small sample sizes, short study durations, and inconsistent protocols regarding probiotic strains and dosages, thereby limiting reproducibility and generalizability [118],[119]. Well-designed, large-scale randomized controlled trials are urgently needed to evaluate the safety, efficacy, and optimal formulations of probiotics, while also accounting for individual microbiome profiles and comorbidities such as ongoing alcohol use and malnutrition [118]. Longitudinal follow-up is essential to determine the durability of microbial shifts and any sustained clinical benefits. In addition, the regulatory landscape for probiotics varies globally, which hampers the development of standardized guidelines and quality control for therapeutic use. Although probiotics are generally regarded as safe, potential adverse effects—including gastrointestinal discomfort and interactions with medications—must be considered and transparently communicated to patients [120]. In patients with cirrhosis, the integrity of the intestinal barrier is often compromised, likely as a consequence of reduced intestinal perfusion due to portal hypertension, diminished bile acid secretion, and gut dysbiosis [121]. These disruptions raise legitimate concerns regarding the potential for probiotic translocation in this vulnerable population. Gram-negative bacteria such as *E. coli* and *Klebsiella* spp., along with enterococci and various *Streptococcus* species, have been shown to translocate to mesenteric lymph nodes, even across histologically intact intestinal mucosa [122]. In contrast, probiotics have only rarely been associated with translocation [122], and they have been reported to inhibit the translocation of pathogenic bacteria and their metabolites to the liver, thereby attenuating hepatic inflammatory responses [31]. Nevertheless, the host's clinical condition remains a critical determinant of whether probiotics exert beneficial or adverse effects. Given the complexity of the human gut microbiome, the multifaceted interactions between probiotics and host physiology, and the strain-specific nature of probiotic effects, further investigation is essential to clarify their therapeutic potential in cirrhosis [120].

## 7. Summary and future research directions

The strong evidence surrounding the relationship between alcohol consumption, GM dysbiosis, and the progression of ALD highlights a critical avenue for therapeutic intervention. Alcohol use not only adversely affects liver health but also disrupts the delicate balance of GM, leading to a series of processes that exacerbate liver inflammation and damage. Probiotics, prebiotics, postbiotics, and synbiotics represent promising strategies for mitigating the detrimental effects of alcohol on liver function by restoring intestinal microbial diversity and reinforcing the gut barrier.

Probiotic supplementation has shown potential to improve liver health by decreasing inflammatory markers, enhancing gut barrier integrity, and reducing levels of harmful gut-derived metabolites such as endotoxins. Prebiotics, alternatively, can promote the growth of beneficial microbial communities, further supporting gut health and liver function. The synergistic use of synbiotics is a multifaceted therapeutic approach that may yield enhanced clinical outcomes for individuals affected by ALD.

Despite these promising findings, many questions remain unresolved. Future research should focus on several key areas:

1. Mechanisms of action: A comprehensive understanding of the specific mechanisms by which probiotics, prebiotics, and synbiotics exert their beneficial effects on the gut-liver axis is essential. Detailed studies are required to elucidate the interactions between microbial metabolites, host immune responses, and liver health.
2. Optimal dosage and administration protocols: Investigating the appropriate doses, formulations, and treatment regimens of these interventions will be critical for maximizing their clinical efficacy. Personalized approaches may enhance treatment success as individual responses to microbial therapies can vary widely.
3. Longitudinal studies: Long-term studies are necessary to assess the durability of the benefits conferred by these therapies and to understand their impact on patients with varying stages of ALD. This research should include assessments of liver function, microbiome composition, and overall health outcomes over extended periods.
4. Clinical trials: Comprehensive clinical trials examining the effects of probiotics and prebiotics in diverse populations with ALD will provide crucial insights into their therapeutic potential. These trials should aim to establish well-defined guidelines for clinical practice, identifying which patient populations may benefit the most from microbiota-targeted therapies.
5. Integration into standard care: Given the central role of gut-derived microbial metabolites in mediating the pathogenesis of ALD, therapeutic strategies aimed at restoring microbial balance and intestinal barrier function offer a promising complement to traditional approaches. Current management of ALD primarily focuses on alcohol cessation, nutritional support, and pharmacologic interventions targeting inflammation and fibrosis. However, these treatments often fail to address the upstream drivers of disease rooted in the gut-liver axis. Among microbiota-targeted interventions, probiotics have garnered significant attention for their potential to modulate gut dysbiosis, enhance intestinal barrier integrity, and attenuate systemic inflammation associated with ALD. Despite these promising findings, the integration of probiotics into ALD treatment regimens necessitates a personalized approach, considering individual microbiome profiles and disease severity. Collaborative research between microbiologists, hepatologists, and nutritionists will be essential to develop comprehensive management strategies that include GM modulation.

By addressing these areas, future research can significantly advance our understanding of ALD and potentially revolutionize its treatment paradigm through microbiota-centered approaches. Increased emphasis on the gut-liver axis and its therapeutic manipulation promises not only to improve liver outcomes but also to contribute to the overall health and well-being of individuals with alcohol-related disorders.

## Use of AI tools declaration

The authors declare they have not used Artificial Intelligence (AI) tools in the creation of this article.
